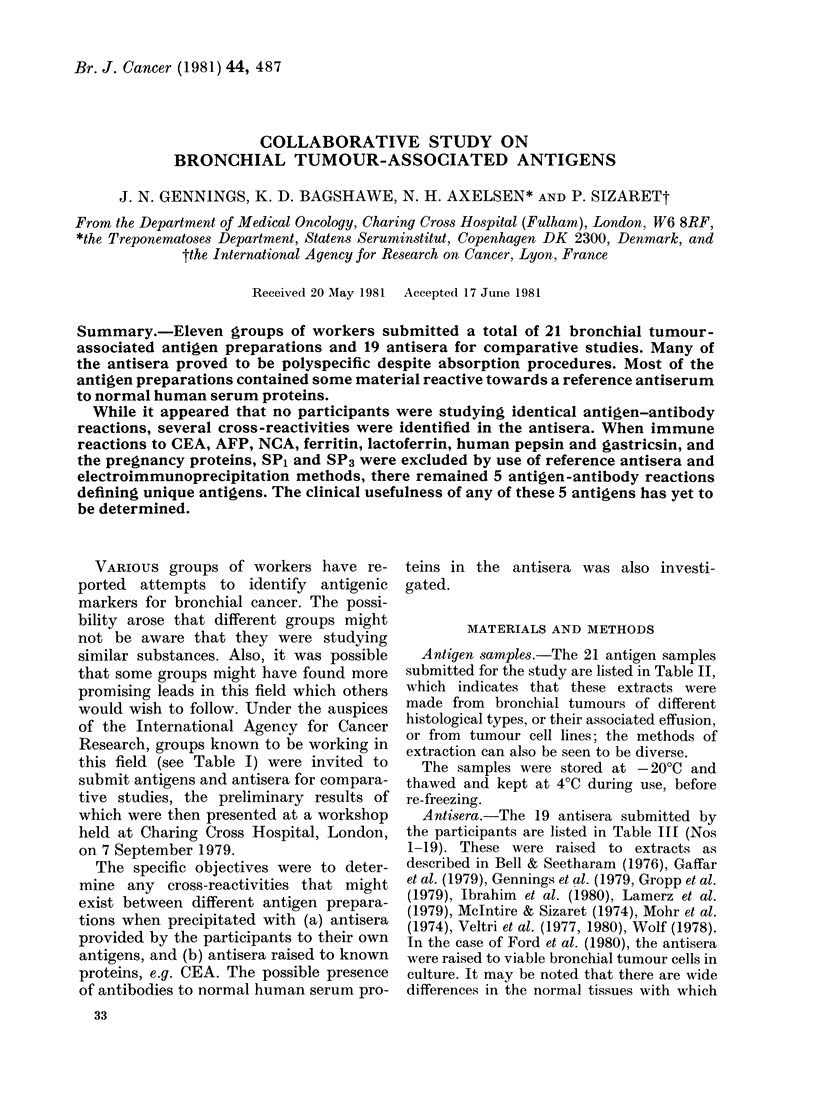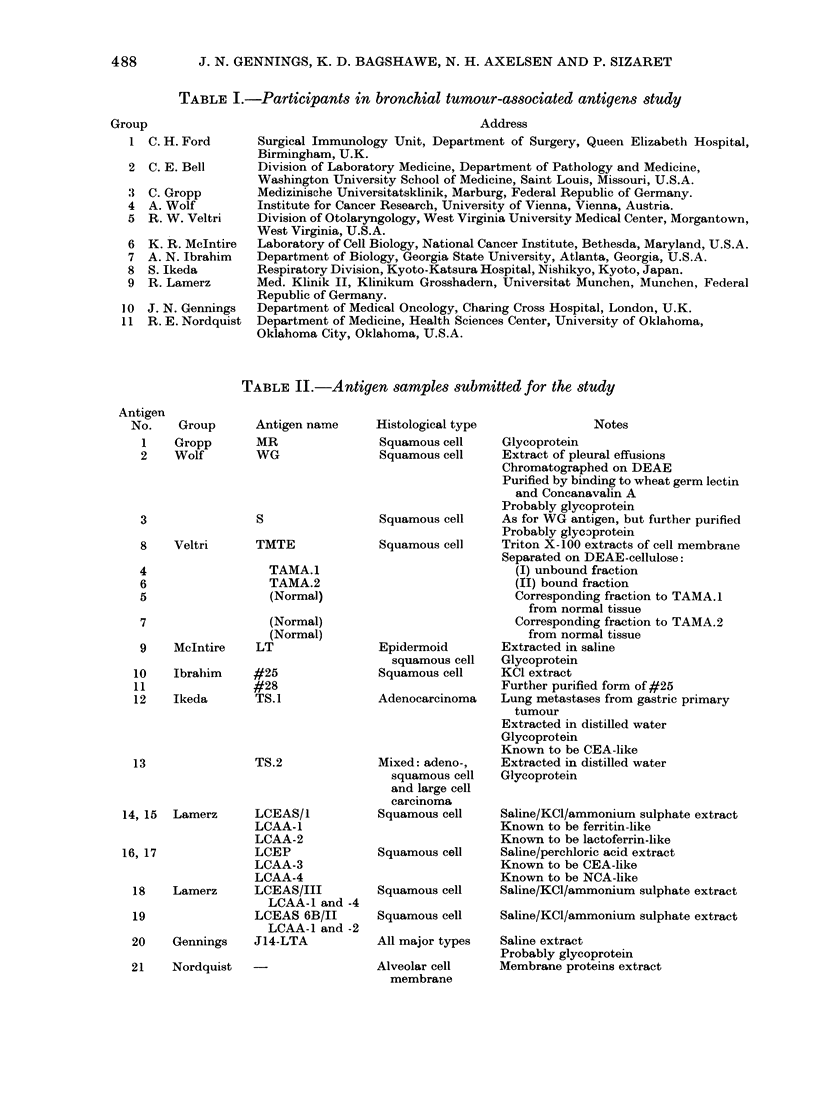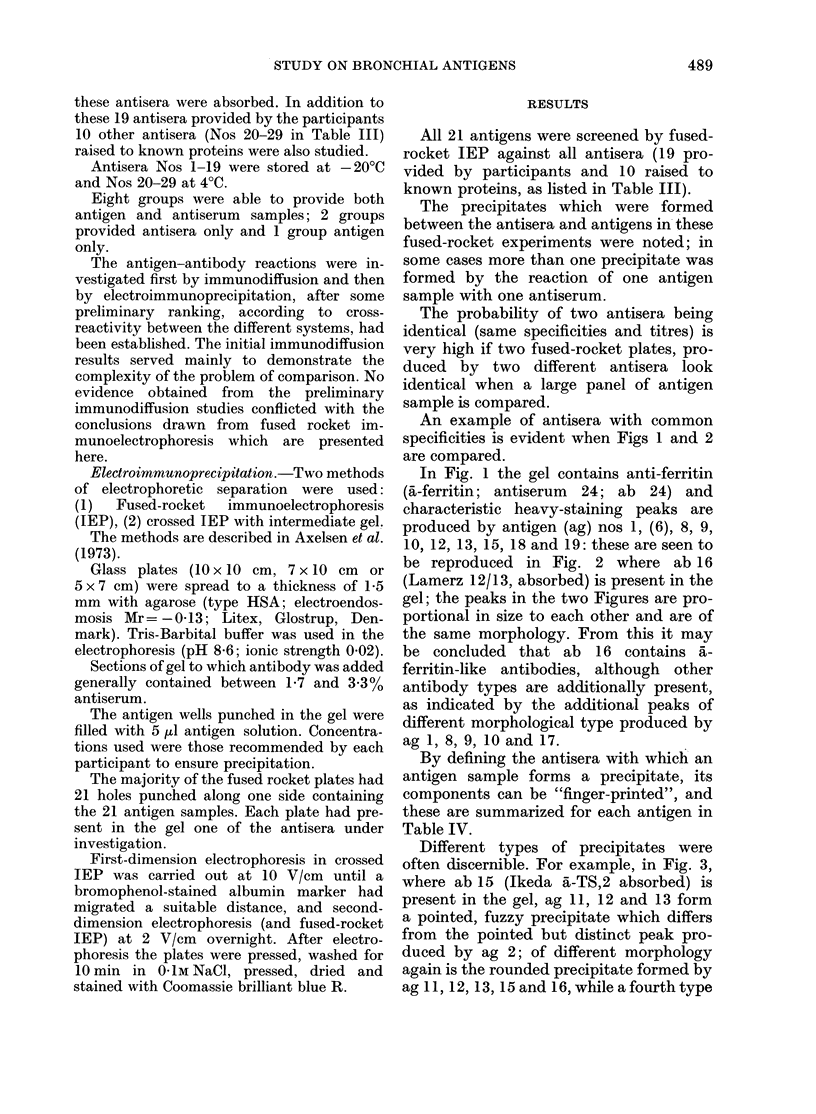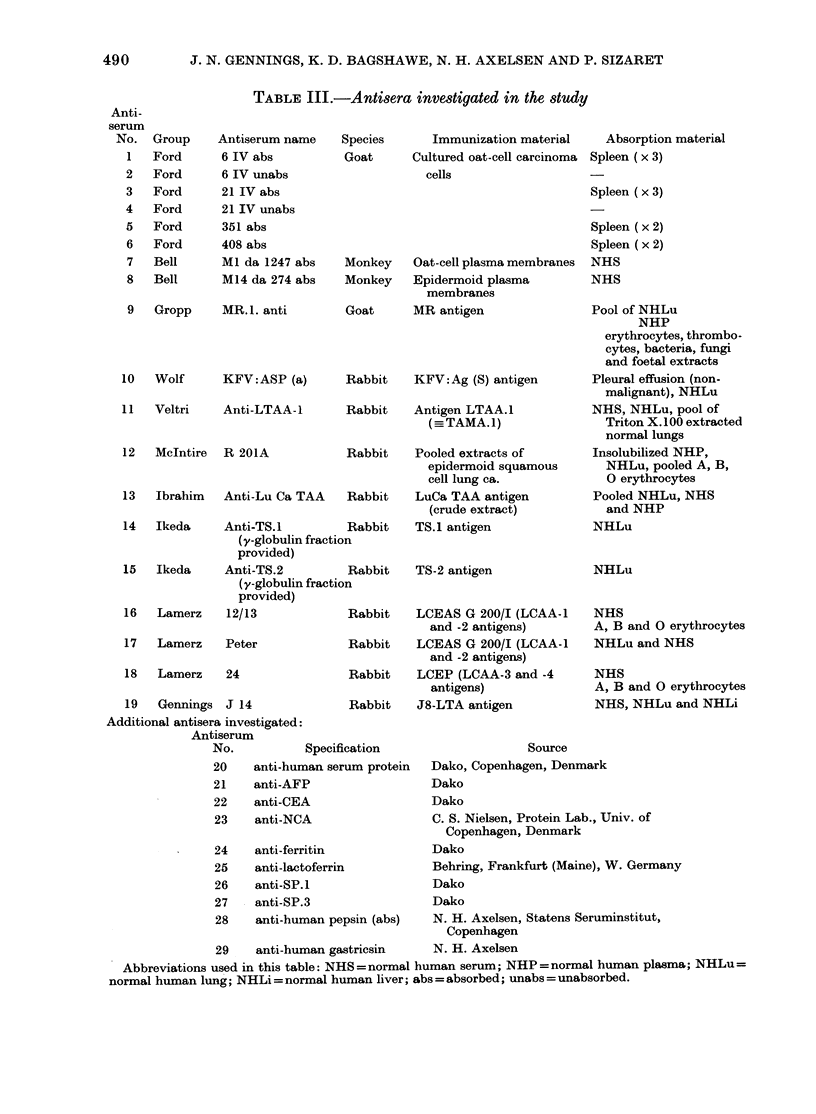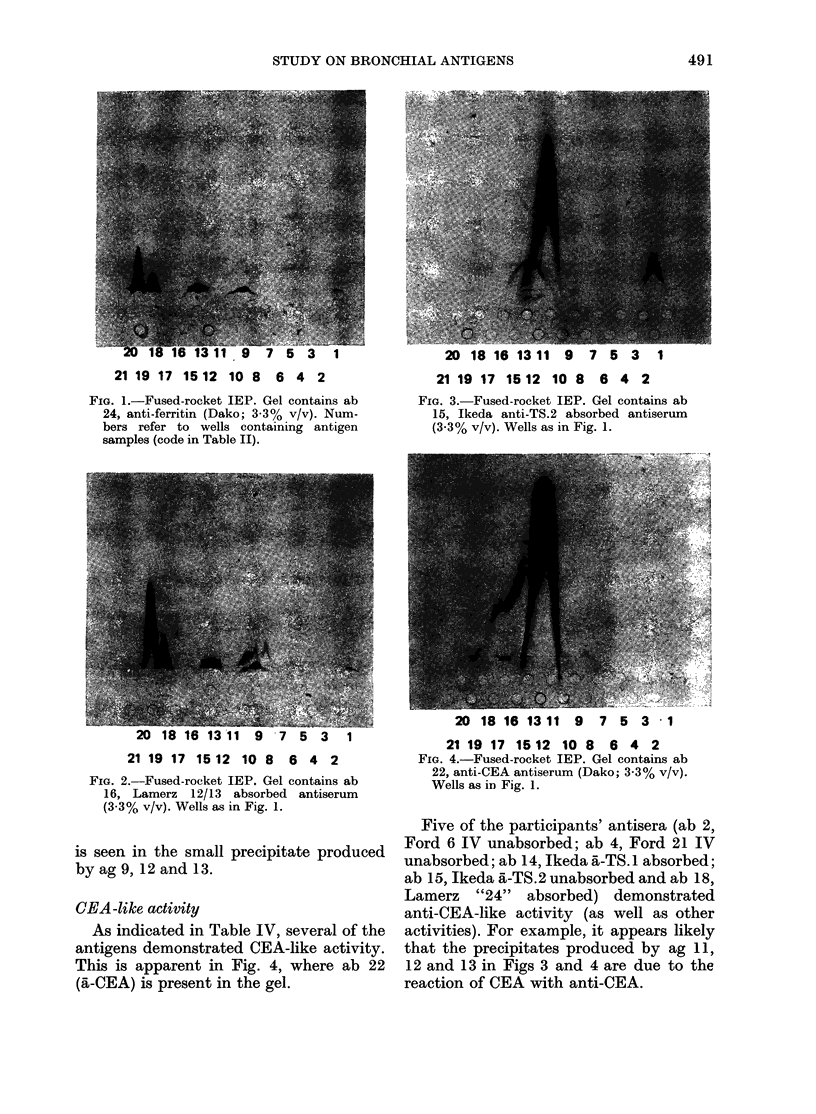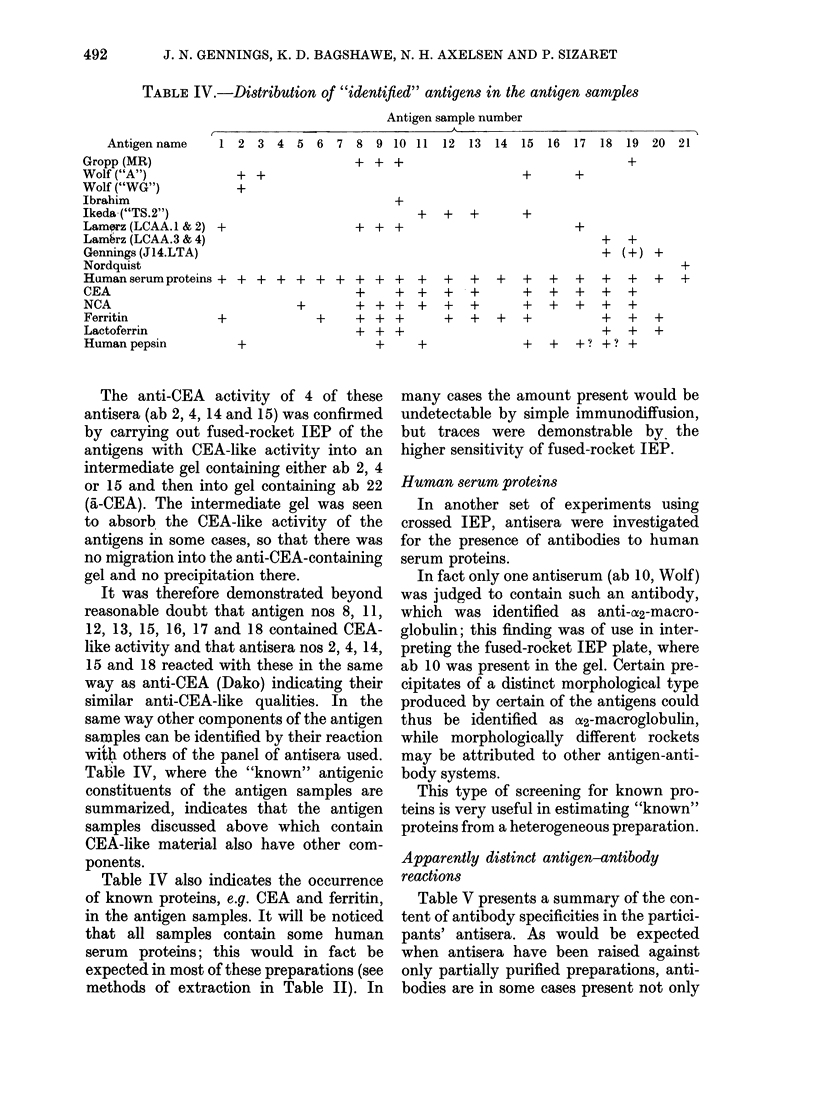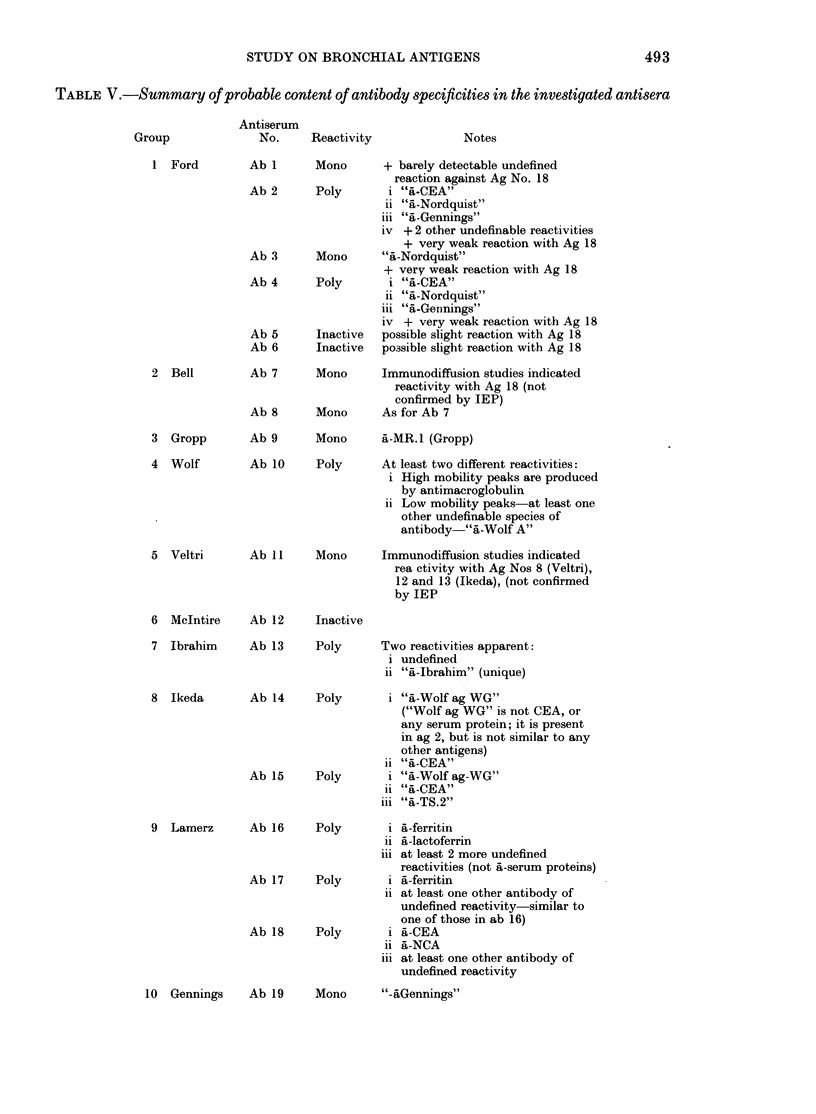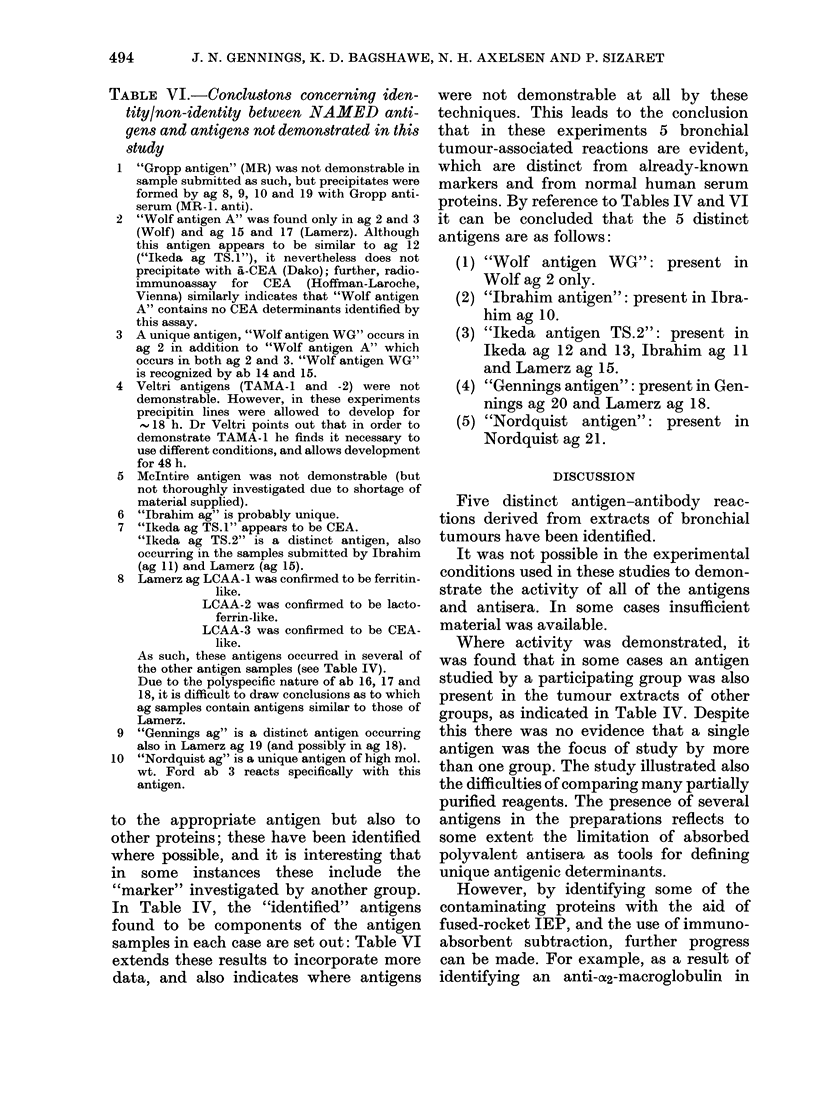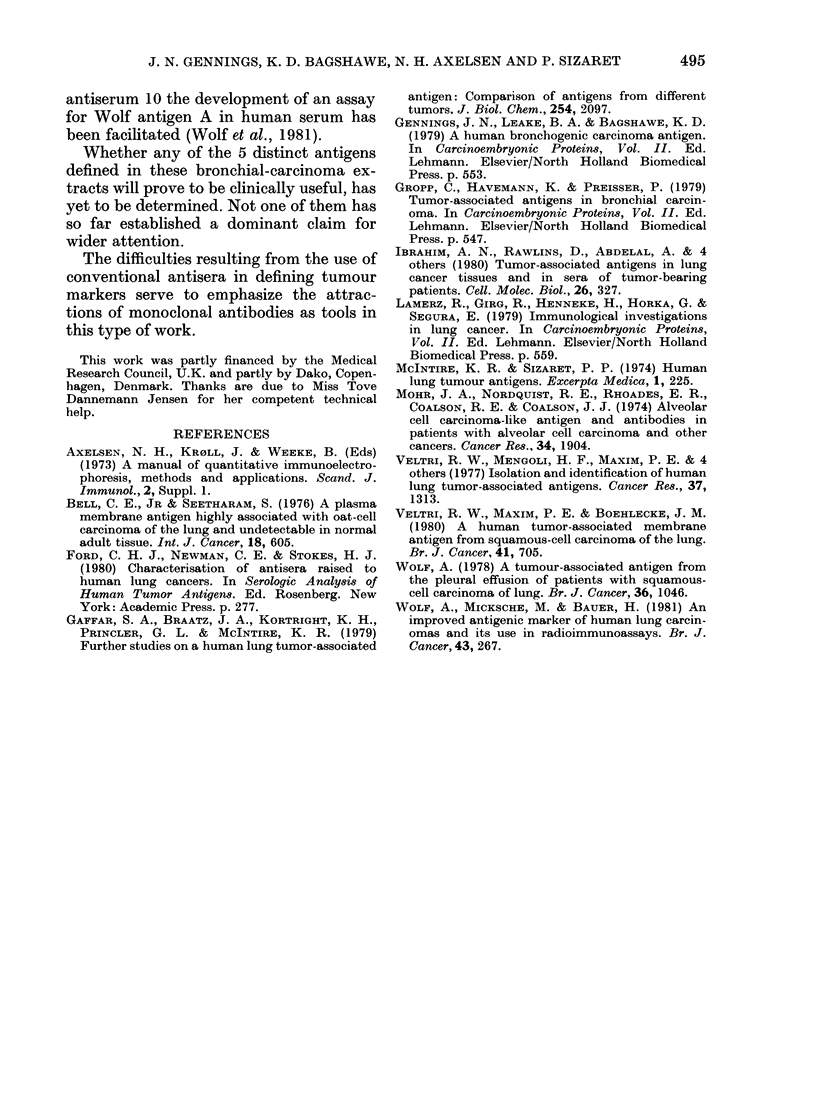# Collaborative study of bronchial tumour-associated antigens.

**DOI:** 10.1038/bjc.1981.216

**Published:** 1981-10

**Authors:** J. N. Gennings, K. D. Bagshawe, N. H. Axelsen, P. Sizaret

## Abstract

**Images:**


*Br. J. Cancer* (1981) **44,** 487

### COLLABORATIVE STUDY ON BRONCHIAL TUMOUR-ASSOCIATED ANTIGENS

J. N. GENNINGS, K. D. BAGSHAWE, N. H. AXELSEN* AND P. SIZARET†

*From the Department of Medical Oncology, Charing Cross Hospital (Fulham), London, W6 8RF, *the Treponematoses Department, Statens Seruminstitut, Copenhagen DK 2300, Denmark, and †the International Agency for Research on Cancer, Lyon, France*

Received 20 May 1981 Accepted 17 June 1981

**Summary.—Eleven groups of workers submitted a total of 21 bronchial tumour-associated antigen preparations and 19 antisera for comparative studies. Many of the antisera proved to be polyspecific despite absorption procedures. Most of the antigen preparations contained some material reactive towards a reference antiserum to normal human serum proteins.**

**While it appeared that no participants were studying identical antigen–antibody reactions, several cross-reactivities were identified in the antisera. When immune reactions to CEA, AFP, NCA, ferritin, lactoferrin, human pepsin and gastricsin, and the pregnancy proteins, $SP_1$ and $SP_3$ were excluded by use of reference antisera and electroimmunoprecipitation methods, there remained 5 antigen-antibody reactions defining unique antigens. The clinical usefulness of any of these 5 antigens has yet to be determined.**

VARIOUS groups of workers have reported attempts to identify antigenic markers for bronchial cancer. The possibility arose that different groups might not be aware that they were studying similar substances. Also, it was possible that some groups might have found more promising leads in this field which others would wish to follow. Under the auspices of the International Agency for Cancer Research, groups known to be working in this field (see Table I) were invited to submit antigens and antisera for comparative studies, the preliminary results of which were then presented at a workshop held at Charing Cross Hospital, London, on 7 September 1979.

The specific objectives were to determine any cross-reactivities that might exist between different antigen preparations when precipitated with (a) antisera provided by the participants to their own antigens, and (b) antisera raised to known proteins, *e.g.* CEA. The possible presence of antibodies to normal human serum proteins in the antisera was also investigated.

### MATERIALS AND METHODS

*Antigen samples.*—The 21 antigen samples submitted for the study are listed in Table II, which indicates that these extracts were made from bronchial tumours of different histological types, or their associated effusion, or from tumour cell lines; the methods of extraction can also be seen to be diverse.

The samples were stored at −20°C and thawed and kept at 4°C during use, before re-freezing.

*Antisera.*—The 19 antisera submitted by the participants are listed in Table III (Nos 1–19). These were raised to extracts as described in Bell & Seetharam (1976), Gaffar *et al.* (1979), Gennings *et al.* (1979, Gropp *et al.* (1979), Ibrahim *et al.* (1980), Lamerz *et al.* (1979), McIntire & Sizaret (1974), Mohr *et al.* (1974), Veltri *et al.* (1977, 1980), Wolf (1978). In the case of Ford *et al.* (1980), the antisera were raised to viable bronchial tumour cells in culture. It may be noted that there are wide differences in the normal tissues with which

TABLE I.—*Participants in bronchial tumour-associated antigens study*

| Group | | Address |
|---|---|---|
| 1 | C. H. Ford | Surgical Immunology Unit, Department of Surgery, Queen Elizabeth Hospital, Birmingham, U.K. |
| 2 | C. E. Bell | Division of Laboratory Medicine, Department of Pathology and Medicine, Washington University School of Medicine, Saint Louis, Missouri, U.S.A. |
| 3 | C. Gropp | Medizinische Universitatsklinik, Marburg, Federal Republic of Germany. |
| 4 | A. Wolf | Institute for Cancer Research, University of Vienna, Vienna, Austria. |
| 5 | R. W. Veltri | Division of Otolaryngology, West Virginia University Medical Center, Morgantown, West Virginia, U.S.A. |
| 6 | K. R. McIntire | Laboratory of Cell Biology, National Cancer Institute, Bethesda, Maryland, U.S.A. |
| 7 | A. N. Ibrahim | Department of Biology, Georgia State University, Atlanta, Georgia, U.S.A. |
| 8 | S. Ikeda | Respiratory Division, Kyoto-Katsura Hospital, Nishikyo, Kyoto, Japan. |
| 9 | R. Lamerz | Med. Klinik II, Klinikum Grosshadern, Universitat Munchen, Munchen, Federal Republic of Germany. |
| 10 | J. N. Gennings | Department of Medical Oncology, Charing Cross Hospital, London, U.K. |
| 11 | R. E. Nordquist | Department of Medicine, Health Sciences Center, University of Oklahoma, Oklahoma City, Oklahoma, U.S.A. |

TABLE II.—*Antigen samples submitted for the study*

| Antigen No. | Group | Antigen name | Histological type | Notes |
|---|---|---|---|---|
| 1 | Gropp | MR | Squamous cell | Glycoprotein |
| 2 | Wolf | WG | Squamous cell | Extract of pleural effusions<br>Chromatographed on DEAE<br>Purified by binding to wheat germ lectin and Concanavalin A<br>Probably glycoprotein |
| 3 | | S | Squamous cell | As for WG antigen, but further purified<br>Probably glycoprotein |
| 8 | Veltri | TMTE | Squamous cell | Triton X-100 extracts of cell membrane<br>Separated on DEAE-cellulose: |
| 4 | | TAMA.1 | | (I) unbound fraction |
| 6 | | TAMA.2 | | (II) bound fraction |
| 5 | | (Normal) | | Corresponding fraction to TAMA.1 from normal tissue |
| 7 | | (Normal)<br>(Normal) | | Corresponding fraction to TAMA.2 from normal tissue |
| 9 | McIntire | LT | Epidermoid squamous cell | Extracted in saline<br>Glycoprotein |
| 10 | Ibrahim | #25 | Squamous cell | KCl extract |
| 11 | | #28 | | Further purified form of #25 |
| 12 | Ikeda | TS.1 | Adenocarcinoma | Lung metastases from gastric primary tumour<br>Extracted in distilled water<br>Glycoprotein<br>Known to be CEA-like |
| 13 | | TS.2 | Mixed: adeno-, squamous cell and large cell carcinoma | Extracted in distilled water<br>Glycoprotein |
| 14, 15 | Lamerz | LCEAS/1<br>LCAA-1<br>LCAA-2 | Squamous cell | Saline/KCl/ammonium sulphate extract<br>Known to be ferritin-like<br>Known to be lactoferrin-like |
| 16, 17 | | LCEP<br>LCAA-3<br>LCAA-4 | Squamous cell | Saline/perchloric acid extract<br>Known to be CEA-like<br>Known to be NCA-like |
| 18 | Lamerz | LCEAS/III<br>LCAA-1 and -4 | Squamous cell | Saline/KCl/ammonium sulphate extract |
| 19 | | LCEAS 6B/II<br>LCAA-1 and -2 | Squamous cell | Saline/KCl/ammonium sulphate extract |
| 20 | Gennings | J14-LTA | All major types | Saline extract<br>Probably glycoprotein |
| 21 | Nordquist | — | Alveolar cell membrane | Membrane proteins extract |

these antisera were absorbed. In addition to these 19 antisera provided by the participants 10 other antisera (Nos 20–29 in Table III) raised to known proteins were also studied.

Antisera Nos 1–19 were stored at −20°C and Nos 20–29 at 4°C.

Eight groups were able to provide both antigen and antiserum samples; 2 groups provided antisera only and 1 group antigen only.

The antigen–antibody reactions were investigated first by immunodiffusion and then by electroimmunoprecipitation, after some preliminary ranking, according to cross-reactivity between the different systems, had been established. The initial immunodiffusion results served mainly to demonstrate the complexity of the problem of comparison. No evidence obtained from the preliminary immunodiffusion studies conflicted with the conclusions drawn from fused rocket immunoelectrophoresis which are presented here.

*Electroimmunoprecipitation.*—Two methods of electrophoretic separation were used: (1) Fused-rocket immunoelectrophoresis (IEP), (2) crossed IEP with intermediate gel.

The methods are described in Axelsen *et al.* (1973).

Glass plates (10 × 10 cm, 7 × 10 cm or 5 × 7 cm) were spread to a thickness of 1·5 mm with agarose (type HSA; electroendosmosis Mr = −0·13; Litex, Glostrup, Denmark). Tris-Barbital buffer was used in the electrophoresis (pH 8·6; ionic strength 0·02).

Sections of gel to which antibody was added generally contained between 1·7 and 3·3% antiserum.

The antigen wells punched in the gel were filled with 5 $\mu$l antigen solution. Concentrations used were those recommended by each participant to ensure precipitation.

The majority of the fused rocket plates had 21 holes punched along one side containing the 21 antigen samples. Each plate had present in the gel one of the antisera under investigation.

First-dimension electrophoresis in crossed IEP was carried out at 10 V/cm until a bromophenol-stained albumin marker had migrated a suitable distance, and second-dimension electrophoresis (and fused-rocket IEP) at 2 V/cm overnight. After electrophoresis the plates were pressed, washed for 10 min in 0·1M NaCl, pressed, dried and stained with Coomassie brilliant blue R.

RESULTS

All 21 antigens were screened by fused-rocket IEP against all antisera (19 provided by participants and 10 raised to known proteins, as listed in Table III).

The precipitates which were formed between the antisera and antigens in these fused-rocket experiments were noted; in some cases more than one precipitate was formed by the reaction of one antigen sample with one antiserum.

The probability of two antisera being identical (same specificities and titres) is very high if two fused-rocket plates, produced by two different antisera look identical when a large panel of antigen sample is compared.

An example of antisera with common specificities is evident when Figs 1 and 2 are compared.

In Fig. 1 the gel contains anti-ferritin (ā-ferritin; antiserum 24; ab 24) and characteristic heavy-staining peaks are produced by antigen (ag) nos 1, (6), 8, 9, 10, 12, 13, 15, 18 and 19: these are seen to be reproduced in Fig. 2 where ab 16 (Lamerz 12/13, absorbed) is present in the gel; the peaks in the two Figures are proportional in size to each other and are of the same morphology. From this it may be concluded that ab 16 contains ā-ferritin-like antibodies, although other antibody types are additionally present, as indicated by the additional peaks of different morphological type produced by ag 1, 8, 9, 10 and 17.

By defining the antisera with which an antigen sample forms a precipitate, its components can be "finger-printed", and these are summarized for each antigen in Table IV.

Different types of precipitates were often discernible. For example, in Fig. 3, where ab 15 (Ikeda ā-TS,2 absorbed) is present in the gel, ag 11, 12 and 13 form a pointed, fuzzy precipitate which differs from the pointed but distinct peak produced by ag 2; of different morphology again is the rounded precipitate formed by ag 11, 12, 13, 15 and 16, while a fourth type

TABLE III.—*Antisera investigated in the study*

| Antiserum No. | Group | Antiserum name | Species | Immunization material | Absorption material |
|---|---|---|---|---|---|
| 1 | Ford | 6 IV abs | Goat | Cultured oat-cell carcinoma | Spleen (× 3) |
| 2 | Ford | 6 IV unabs | | cells | — |
| 3 | Ford | 21 IV abs | | | Spleen (× 3) |
| 4 | Ford | 21 IV unabs | | | — |
| 5 | Ford | 351 abs | | | Spleen (× 2) |
| 6 | Ford | 408 abs | | | Spleen (× 2) |
| 7 | Bell | M1 da 1247 abs | Monkey | Oat-cell plasma membranes | NHS |
| 8 | Bell | M14 da 274 abs | Monkey | Epidermoid plasma membranes | NHS |
| 9 | Gropp | MR.1. anti | Goat | MR antigen | Pool of NHLu NHP erythrocytes, thrombocytes, bacteria, fungi and foetal extracts |
| 10 | Wolf | KFV:ASP (a) | Rabbit | KFV:Ag (S) antigen | Pleural effusion (non-malignant), NHLu |
| 11 | Veltri | Anti-LTAA-1 | Rabbit | Antigen LTAA.1 (≡TAMA.1) | NHS, NHLu, pool of Triton X.100 extracted normal lungs |
| 12 | McIntire | R 201A | Rabbit | Pooled extracts of epidermoid squamous cell lung ca. | Insolubilized NHP, NHLu, pooled A, B, O erythrocytes |
| 13 | Ibrahim | Anti-Lu Ca TAA | Rabbit | LuCa TAA antigen (crude extract) | Pooled NHLu, NHS and NHP |
| 14 | Ikeda | Anti-TS.1 (γ-globulin fraction provided) | Rabbit | TS.1 antigen | NHLu |
| 15 | Ikeda | Anti-TS.2 (γ-globulin fraction provided) | Rabbit | TS-2 antigen | NHLu |
| 16 | Lamerz | 12/13 | Rabbit | LCEAS G 200/I (LCAA-1 and -2 antigens) | NHS A, B and O erythrocytes |
| 17 | Lamerz | Peter | Rabbit | LCEAS G 200/I (LCAA-1 and -2 antigens) | NHLu and NHS |
| 18 | Lamerz | 24 | Rabbit | LCEP (LCAA-3 and -4 antigens) | NHS A, B and O erythrocytes |
| 19 | Gennings | J 14 | Rabbit | J8-LTA antigen | NHS, NHLu and NHLi |

Additional antisera investigated:

| Antiserum No. | Specification | Source |
|---|---|---|
| 20 | anti-human serum protein | Dako, Copenhagen, Denmark |
| 21 | anti-AFP | Dako |
| 22 | anti-CEA | Dako |
| 23 | anti-NCA | C. S. Nielsen, Protein Lab., Univ. of Copenhagen, Denmark |
| 24 | anti-ferritin | Dako |
| 25 | anti-lactoferrin | Behring, Frankfurt (Maine), W. Germany |
| 26 | anti-SP.1 | Dako |
| 27 | anti-SP.3 | Dako |
| 28 | anti-human pepsin (abs) | N. H. Axelsen, Statens Seruminstitut, Copenhagen |
| 29 | anti-human gastricsin | N. H. Axelsen |

Abbreviations used in this table: NHS = normal human serum; NHP = normal human plasma; NHLu = normal human lung; NHLi = normal human liver; abs = absorbed; unabs = unabsorbed.

Fig. 1.—Fused-rocket IEP. Gel contains ab 24, anti-ferritin (Dako; 3·3% v/v). Numbers refer to wells containing antigen samples (code in Table II).

Fig. 2.—Fused-rocket IEP. Gel contains ab 16, Lamerz 12/13 absorbed antiserum (3·3% v/v). Wells as in Fig. 1.

is seen in the small precipitate produced by ag 9, 12 and 13.

*CEA-like activity*

As indicated in Table IV, several of the antigens demonstrated CEA-like activity. This is apparent in Fig. 4, where ab 22 (ā-CEA) is present in the gel.

Fig. 3.—Fused-rocket IEP. Gel contains ab 15, Ikeda anti-TS.2 absorbed antiserum (3·3% v/v). Wells as in Fig. 1.

Fig. 4.—Fused-rocket IEP. Gel contains ab 22, anti-CEA antiserum (Dako; 3·3% v/v). Wells as in Fig. 1.

Five of the participants' antisera (ab 2, Ford 6 IV unabsorbed; ab 4, Ford 21 IV unabsorbed; ab 14, Ikeda ā-TS.1 absorbed; ab 15, Ikeda ā-TS.2 unabsorbed and ab 18, Lamerz "24" absorbed) demonstrated anti-CEA-like activity (as well as other activities). For example, it appears likely that the precipitates produced by ag 11, 12 and 13 in Figs 3 and 4 are due to the reaction of CEA with anti-CEA.

TABLE IV.—*Distribution of "identified" antigens in the antigen samples*

| Antigen name | Antigen sample number | | | | | | | | | | | | | | | | | | | | |
|---|---|---|---|---|---|---|---|---|---|---|---|---|---|---|---|---|---|---|---|---|---|
| | 1 | 2 | 3 | 4 | 5 | 6 | 7 | 8 | 9 | 10 | 11 | 12 | 13 | 14 | 15 | 16 | 17 | 18 | 19 | 20 | 21 |
| Gropp (MR) | | | | | | | | + | + | + | | | | | | | | | + | | |
| Wolf ("A") | | + | + | | | | | | | | | | | | + | | + | | | | |
| Wolf ("WG") | | + | | | | | | | | | | | | | | | | | | | |
| Ibrahim | | | | | | | | | | + | | | | | | | | | | | |
| Ikeda ("TS.2") | | | | | | | | | | | + | + | + | | + | | | | | | |
| Lamerz (LCAA.1 & 2) | + | | | | | | | + | + | + | | | | | | | + | | | | |
| Lamerz (LCAA.3 & 4) | | | | | | | | | | | | | | | | | | + | + | | |
| Gennings (J14.LTA) | | | | | | | | | | | | | | | | | | + | (+) | + | |
| Nordquist | | | | | | | | | | | | | | | | | | | | | + |
| Human serum proteins | + | + | + | + | + | + | + | + | + | + | + | + | + | + | + | + | + | + | + | + | + |
| CEA | | | | | | | | + | | + | + | + | + | | + | + | + | + | + | | |
| NCA | | | | | + | | | + | + | + | + | + | + | | + | + | + | + | + | | |
| Ferritin | + | | | | | + | | + | + | + | | + | + | + | + | | | + | + | + | |
| Lactoferrin | | | | | | | | + | + | + | | | | | | | | + | + | + | |
| Human pepsin | | + | | | | | | | + | | + | | | | + | + | +? | +? | + | | |

The anti-CEA activity of 4 of these antisera (ab 2, 4, 14 and 15) was confirmed by carrying out fused-rocket IEP of the antigens with CEA-like activity into an intermediate gel containing either ab 2, 4 or 15 and then into gel containing ab 22 (ā-CEA). The intermediate gel was seen to absorb the CEA-like activity of the antigens in some cases, so that there was no migration into the anti-CEA-containing gel and no precipitation there.

It was therefore demonstrated beyond reasonable doubt that antigen nos 8, 11, 12, 13, 15, 16, 17 and 18 contained CEA-like activity and that antisera nos 2, 4, 14, 15 and 18 reacted with these in the same way as anti-CEA (Dako) indicating their similar anti-CEA-like qualities. In the same way other components of the antigen samples can be identified by their reaction with others of the panel of antisera used. Table IV, where the "known" antigenic constituents of the antigen samples are summarized, indicates that the antigen samples discussed above which contain CEA-like material also have other components.

Table IV also indicates the occurrence of known proteins, *e.g.* CEA and ferritin, in the antigen samples. It will be noticed that all samples contain some human serum proteins; this would in fact be expected in most of these preparations (see methods of extraction in Table II). In many cases the amount present would be undetectable by simple immunodiffusion, but traces were demonstrable by the higher sensitivity of fused-rocket IEP.

### *Human serum proteins*

In another set of experiments using crossed IEP, antisera were investigated for the presence of antibodies to human serum proteins.

In fact only one antiserum (ab 10, Wolf) was judged to contain such an antibody, which was identified as anti-$\alpha_2$-macroglobulin; this finding was of use in interpreting the fused-rocket IEP plate, where ab 10 was present in the gel. Certain precipitates of a distinct morphological type produced by certain of the antigens could thus be identified as $\alpha_2$-macroglobulin, while morphologically different rockets may be attributed to other antigen-antibody systems.

This type of screening for known proteins is very useful in estimating "known" proteins from a heterogeneous preparation.

### *Apparently distinct antigen–antibody reactions*

Table V presents a summary of the content of antibody specificities in the participants' antisera. As would be expected when antisera have been raised against only partially purified preparations, antibodies are in some cases present not only

TABLE V.—*Summary of probable content of antibody specificities in the investigated antisera*

| Group | | Antiserum No. | Reactivity | Notes |
|---|---|---|---|---|
| 1 | Ford | Ab 1 | Mono | + barely detectable undefined reaction against Ag No. 18 |
| | | Ab 2 | Poly | i "ā-CEA"<br>ii "ā-Nordquist"<br>iii "ā-Gennings"<br>iv +2 other undefinable reactivities + very weak reaction with Ag 18 |
| | | Ab 3 | Mono | "ā-Nordquist"<br>+ very weak reaction with Ag 18 |
| | | Ab 4 | Poly | i "ā-CEA"<br>ii "ā-Nordquist"<br>iii "ā-Gennings"<br>iv + very weak reaction with Ag 18 |
| | | Ab 5 | Inactive | possible slight reaction with Ag 18 |
| | | Ab 6 | Inactive | possible slight reaction with Ag 18 |
| 2 | Bell | Ab 7 | Mono | Immunodiffusion studies indicated reactivity with Ag 18 (not confirmed by IEP) |
| | | Ab 8 | Mono | As for Ab 7 |
| 3 | Gropp | Ab 9 | Mono | ā-MR.1 (Gropp) |
| 4 | Wolf | Ab 10 | Poly | At least two different reactivities:<br>i High mobility peaks are produced by antimacroglobulin<br>ii Low mobility peaks—at least one other undefinable species of antibody—"ā-Wolf A" |
| 5 | Veltri | Ab 11 | Mono | Immunodiffusion studies indicated rea ctivity with Ag Nos 8 (Veltri), 12 and 13 (Ikeda), (not confirmed by IEP |
| 6 | McIntire | Ab 12 | Inactive | |
| 7 | Ibrahim | Ab 13 | Poly | Two reactivities apparent:<br>i undefined<br>ii "ā-Ibrahim" (unique) |
| 8 | Ikeda | Ab 14 | Poly | i "ā-Wolf ag WG" ("Wolf ag WG" is not CEA, or any serum protein; it is present in ag 2, but is not similar to any other antigens)<br>ii "ā-CEA" |
| | | Ab 15 | Poly | i "ā-Wolf ag-WG"<br>ii "ā-CEA"<br>iii "ā-TS.2" |
| 9 | Lamerz | Ab 16 | Poly | i ā-ferritin<br>ii ā-lactoferrin<br>iii at least 2 more undefined reactivities (not ā-serum proteins) |
| | | Ab 17 | Poly | i ā-ferritin<br>ii at least one other antibody of undefined reactivity—similar to one of those in ab 16) |
| | | Ab 18 | Poly | i ā-CEA<br>ii ā-NCA<br>iii at least one other antibody of undefined reactivity |
| 10 | Gennings | Ab 19 | Mono | "-āGennings" |

TABLE VI.—*Conclusions concerning identity/non-identity between NAMED antigens and antigens not demonstrated in this study*

1 "Gropp antigen" (MR) was not demonstrable in sample submitted as such, but precipitates were formed by ag 8, 9, 10 and 19 with Gropp antiserum (MR-1. anti).
2 "Wolf antigen A" was found only in ag 2 and 3 (Wolf) and ag 15 and 17 (Lamerz). Although this antigen appears to be similar to ag 12 ("Ikeda ag TS.1"), it nevertheless does not precipitate with ā-CEA (Dako); further, radioimmunoassay for CEA (Hoffman-Laroche, Vienna) similarly indicates that "Wolf antigen A" contains no CEA determinants identified by this assay.
3 A unique antigen, "Wolf antigen WG" occurs in ag 2 in addition to "Wolf antigen A" which occurs in both ag 2 and 3. "Wolf antigen WG" is recognized by ab 14 and 15.
4 Veltri antigens (TAMA-1 and -2) were not demonstrable. However, in these experiments precipitin lines were allowed to develop for ~18 h. Dr Veltri points out that in order to demonstrate TAMA-1 he finds it necessary to use different conditions, and allows development for 48 h.
5 McIntire antigen was not demonstrable (but not thoroughly investigated due to shortage of material supplied).
6 "Ibrahim ag" is probably unique.
7 "Ikeda ag TS.1" appears to be CEA.
"Ikeda ag TS.2" is a distinct antigen, also occurring in the samples submitted by Ibrahim (ag 11) and Lamerz (ag 15).
8 Lamerz ag LCAA-1 was confirmed to be ferritin-like.
LCAA-2 was confirmed to be lacto-ferrin-like.
LCAA-3 was confirmed to be CEA-like.
As such, these antigens occurred in several of the other antigen samples (see Table IV).
Due to the polyspecific nature of ab 16, 17 and 18, it is difficult to draw conclusions as to which ag samples contain antigens similar to those of Lamerz.
9 "Gennings ag" is a distinct antigen occurring also in Lamerz ag 19 (and possibly in ag 18).
10 "Nordquist ag" is a unique antigen of high mol. wt. Ford ab 3 reacts specifically with this antigen.

to the appropriate antigen but also to other proteins; these have been identified where possible, and it is interesting that in some instances these include the "marker" investigated by another group. In Table IV, the "identified" antigens found to be components of the antigen samples in each case are set out: Table VI extends these results to incorporate more data, and also indicates where antigens were not demonstrable at all by these techniques. This leads to the conclusion that in these experiments 5 bronchial tumour-associated reactions are evident, which are distinct from already-known markers and from normal human serum proteins. By reference to Tables IV and VI it can be concluded that the 5 distinct antigens are as follows:

(1) "Wolf antigen WG": present in Wolf ag 2 only.
(2) "Ibrahim antigen": present in Ibrahim ag 10.
(3) "Ikeda antigen TS.2": present in Ikeda ag 12 and 13, Ibrahim ag 11 and Lamerz ag 15.
(4) "Gennings antigen": present in Gennings ag 20 and Lamerz ag 18.
(5) "Nordquist antigen": present in Nordquist ag 21.

### DISCUSSION

Five distinct antigen–antibody reactions derived from extracts of bronchial tumours have been identified.

It was not possible in the experimental conditions used in these studies to demonstrate the activity of all of the antigens and antisera. In some cases insufficient material was available.

Where activity was demonstrated, it was found that in some cases an antigen studied by a participating group was also present in the tumour extracts of other groups, as indicated in Table IV. Despite this there was no evidence that a single antigen was the focus of study by more than one group. The study illustrated also the difficulties of comparing many partially purified reagents. The presence of several antigens in the preparations reflects to some extent the limitation of absorbed polyvalent antisera as tools for defining unique antigenic determinants.

However, by identifying some of the contaminating proteins with the aid of fused-rocket IEP, and the use of immunoabsorbent subtraction, further progress can be made. For example, as a result of identifying an anti-$\alpha_2$-macroglobulin in antiserum 10 the development of an assay for Wolf antigen A in human serum has been facilitated (Wolf *et al.*, 1981).

Whether any of the 5 distinct antigens defined in these bronchial-carcinoma extracts will prove to be clinically useful, has yet to be determined. Not one of them has so far established a dominant claim for wider attention.

The difficulties resulting from the use of conventional antisera in defining tumour markers serve to emphasize the attractions of monoclonal antibodies as tools in this type of work.

This work was partly financed by the Medical Research Council, U.K. and partly by Dako, Copenhagen, Denmark. Thanks are due to Miss Tove Dannemann Jensen for her competent technical help.

REFERENCES

AXELSEN, N. H., KRØLL, J. & WEEKE, B. (Eds) (1973) A manual of quantitative immunoelectrophoresis, methods and applications. *Scand. J. Immunol.*, **2**, Suppl. 1.

BELL, C. E., JR & SEETHARAM, S. (1976) A plasma membrane antigen highly associated with oat-cell carcinoma of the lung and undetectable in normal adult tissue. *Int. J. Cancer*, **18**, 605.

FORD, C. H. J., NEWMAN, C. E. & STOKES, H. J. (1980) Characterisation of antisera raised to human lung cancers. In *Serologic Analysis of Human Tumor Antigens*. Ed. Rosenberg. New York: Academic Press. p. 277.

GAFFAR, S. A., BRAATZ, J. A., KORTRIGHT, K. H., PRINCLER, G. L. & McINTIRE, K. R. (1979) Further studies on a human lung tumor-associated antigen: Comparison of antigens from different tumors. *J. Biol. Chem.*, **254**, 2097.

GENNINGS, J. N., LEAKE, B. A. & BAGSHAWE, K. D. (1979) A human bronchogenic carcinoma antigen. In *Carcinoembryonic Proteins, Vol. II*. Ed. Lehmann. Elsevier/North Holland Biomedical Press. p. 553.

GROPP, C., HAVEMANN, K. & PREISSER, P. (1979) Tumor-associated antigens in bronchial carcinoma. In *Carcinoembryonic Proteins, Vol. II*. Ed. Lehmann. Elsevier/North Holland Biomedical Press. p. 547.

IBRAHIM, A. N., RAWLINS, D., ABDELAL, A. & 4 others (1980) Tumor-associated antigens in lung cancer tissues and in sera of tumor-bearing patients. *Cell. Molec. Biol.*, **26**, 327.

LAMERZ, R., GIRG, R., HENNEKE, H., HORKA, G. & SEGURA, E. (1979) Immunological investigations in lung cancer. In *Carcinoembryonic Proteins, Vol. II*. Ed. Lehmann. Elsevier/North Holland Biomedical Press. p. 559.

McINTIRE, K. R. & SIZARET, P. P. (1974) Human lung tumour antigens. *Excerpta Medica*, **1**, 225.

MOHR, J. A., NORDQUIST, R. E., RHOADES, E. R., COALSON, R. E. & COALSON, J. J. (1974) Alveolar cell carcinoma-like antigen and antibodies in patients with alveolar cell carcinoma and other cancers. *Cancer Res.*, **34**, 1904.

VELTRI, R. W., MENGOLI, H. F., MAXIM, P. E. & 4 others (1977) Isolation and identification of human lung tumor-associated antigens. *Cancer Res.*, **37**, 1313.

VELTRI, R. W., MAXIM, P. E. & BOEHLECKE, J. M. (1980) A human tumor-associated membrane antigen from squamous-cell carcinoma of the lung. *Br. J. Cancer*, **41**, 705.

WOLF, A. (1978) A tumour-associated antigen from the pleural effusion of patients with squamous-cell carcinoma of lung. *Br. J. Cancer*, **36**, 1046.

WOLF, A., MICKSCHE, M. & BAUER, H. (1981) An improved antigenic marker of human lung carcinomas and its use in radioimmunoassays. *Br. J. Cancer*, **43**, 267.